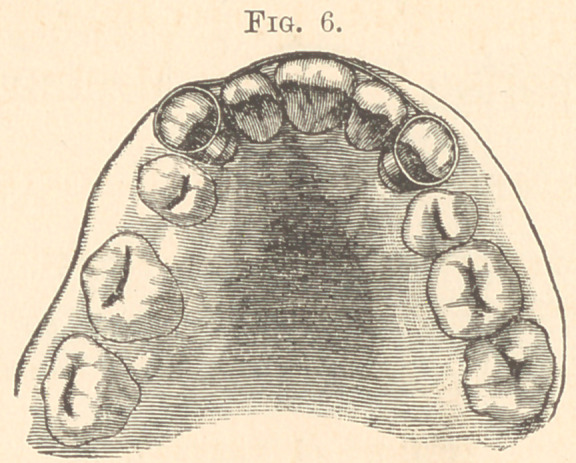# Physiology of Tooth-Movement in Regulating

**Published:** 1889-07

**Authors:** S. H. Guilford


### PHYSIOLOGY OF TOOTH-MOVEMENT IN REGULATING.[1]

BY S. H. GUILFORD, D.D.S., PH.D.

In changing the position of teeth in the act of regulating, the surrounding tissues, both hard and soft, are largely involved. In order, therefore, to properly comprehend the philosophy of tooth-movement it is necessary to understand the structural character of these tissues and the physiological changes that take place in them while a tooth is being moved.

*The Alveolar Process.*—This process, as its name implies, is not a separate and distinct bone, but an outgrowth from another. It is a provisional structure designed to support the teeth in position and afford lodgement for the nutrient vessels leading to them. It is formed upon the body of the bones of the jaw as the teeth are developed, growing with them until they are fully formed, and then remaining while they remain.

When the teeth are lost, there being no longer any special use for it, most of this process is absorbed and carried away. In early infancy little alveolar structure exists, but it is formed co-ordinately with the growth of the deciduous teeth, and remains during the period of their retention. Should they be lost before their successors are ready to appear, the process will be entirely removed by absorption, and a new one be formed for the accommodation of the permanent teeth. Where, however, the deciduous teeth are gradually shed to make way for their successors, the process is not entirely absorbed, the basal and unabsorbed portion serving as a foundation upon which the new alveoli are formed.

The process consists of an outer and inner plate united at intervals by septa, thus forming the alveoli for the accommodation of the roots of the teeth. In structure the process is not compact, but open and spongy, somewhat resembling the cancellated structure of the diploe of the bones of the cranium or the inner portion of the shafts of long bones. Its outer or cortical layer is denser and harder than the inner portion. Its cellular structure, while giving it sufficient firmness to support the teeth in their positions, affords opportunity for the lodgement and passage of the vessels of nutrition with which it is so bountifully supplied.

---

[1] Read at the semi-annual meeting of the Massachusetts Dental Society, Boston, June 6, 1889.

Owing to its peculiar structure and its great vascularity, it is readily resorbed under the stimulus of pressure, and again readily reproduced behind the moving tooth.

*The Teeth.*—Of the teeth themselves but little need be said. All are more or less familiar with their number, shape, position, and structure. Being the hardest structures of the human body, the application of any force necessary to move them will not injuriously affect them so far as their hard tissues are concerned.

In the moving of teeth, the fact must not be overlooked that while the crown may be moved considerably, the movement becomes less and less along the line of the root, so that the apex is moved but little. This is due to the fact that force can only be applied to the crown, while the apex remains almost a fixed point or fulcrum. In the movement of a tooth, therefore, whether inward or outward, forward or backward, the crown describes the arc of a circle, the centre of which is near the apex of the root.

Teeth with single and short roots can be moved more readily than those with long and many roots, for the reason that in the former case there will be less resistance to be overcome.

*The Pulp.*—The pulp is the formative organ of the tooth, and after calcification is complete, it remains as the principal source of nutrient supply for the dentinal tissues, especially the dentine.

It is composed of fibrous connective tissue, containing a delicate system of lymphatics, together with numerous nerve-filaments, which enter through the apical foramen. Ramifications of minute blood-vessels are noticeable throughout its whole extent, giving color to the organ, and constituting its vascular system.

It bears an important relation to the teeth in their movement, since it may be devitalized through imprudence or lack of care. Before calcification of the teeth has been completed the apical foramen is large and easily accommodates the pulp where it enters the tooth. After calcification is complete, the apical foramen is small, and the pulp at this point is in consequence greatly reduced in size. In the movement of the teeth there is often a slight mechanical constriction of the pulp at the apex due to the tipping of the tooth in moving. If the movement be rapid in teeth fully calcified (after the sixteenth or eighteenth year), this constriction may be so great as to cause the death of the pulp through strangulation. Before complete calcification this is not likely to occur, from the fact that when the foramen is large the pulp has more space for its accommodation.

In the movement of a tooth in the direction of its length the pulp may also become devitalized through excessive stretching. This has occurred at times in drawing down into line a tooth that has been retarded in eruption. In all such cases care must be exercised and the movement be conducted slowly.

*The Pericementum.*—The pericementum—or peridental membrane—is that tissue which envelops the root of the tooth and fills the space intervening between it and the alveolar wall. It is a tough, strong membrane, composed mainly of fibrous connective tissue, permeated with blood-vessels and nerve-fibres, and containing traces of a lymphatic system.

It is strongly adherent to the alveolar wall of the socket, on the one hand, and to the cementum of the tooth, on the other; its adherence being due to the extension of its fibres into both the bone and the cementum. These fibres, according to Professor Black,[1] "are wholly of the white or inelastic connective-tissue variety," and the apparent elasticity of the membrane is due to the passage of the fibres from cementum to alveolus often in an oblique direction, in such a way as to "swing the tooth in its socket."

This membrane is the formative organ of the cementum of the tooth, and also assists in building the walls of the alveoli.

The cells concerned in the building of the bony walls are known as osteoblasts, and those forming the cementum are designated cementoblasts. After these cells have performed their normal function, they become encapsuled, and form part of the tissue they were instrumental in building.

When re-formation of tissue is demanded, as in the thickening of the alveolar wall or in enlarging the normal amount of cementum at various points under certain conditions, new cells are originated to perform the work. In the moving of a tooth the activity of these new cells is at once manifested in the formation of alveolar tissue to fill the space caused by the advancing tooth.

Beside these cells of construction and repair the pericementum also contains cells that might well be called *cells of destruction.* They are the osteoclasts or cementoclasts, and their function is to break down or absorb the cemental or osseous tissues when nature calls for such action.

In the correction of irregularities, these cells perform valuable service in removing bony tissue in front of the moving tooth.

The pericementum is thickest in childhood, when the sockets or alveoli are of necessity considerably larger than the roots of the

[1] *Dental Review*, vol. i. p. 240.

teeth which they contain. With advancing age both cementum and the alveolar walls are increased in thickness by slow but continuous growth until the pericementum is greatly reduced in thickness, and, in consequence, the diameter of the roots more nearly approximates that of the alveoli or sockets.

The pericementum possesses a variety of functions not often met with in any single tissue of the human system.

It retains the tooth in its socket and acts as a cushion to prevent injury to the adjoining bony structures from hard and violent concussions to which the teeth are sometimes subjected.

It affords accommodation for numerous blood-vessels, which supply the teeth with nutrient material, and for the branches of nerves which constitute it the sensory organ of the tooth, so far as tactile impress is concerned.

It is the organ of construction and repair of both cementum and alveolar wall, and is also, on occasion, the organ of destruction of either or both of these tissues.

Its great importance in the moving of teeth is shown in the fact that without its services teeth could not be altered in their positions without serious injury to themselves or adjoining parts, and if so moved would be useless, because they could not possibly be made firm in their new positions. In other words, the regulation of teeth would be a physical impossibility without the important services rendered by this peridental membrane.

*Physiological Action in the Movement of a Tooth.*—When force is exerted upon a tooth for the purpose of moving it, the first effect produced is the compression of the pericementum between the tooth and alveolar wall on the advancing side and the stretching of the same membrane on the opposite side. In the compression of the membrane the blood-supply is partly cut off, and the nerves, by their irritation, create a sensation of pain which is soon obliterated by the semi-paralysis brought about by continued pressure. At the same time, this irritation stimulates and hastens the development of the osteoclasts, which at once begin the work of breaking down and absorbing that portion of the alveolus pressed upon.

Bony tissue thus being removed, accommodation is made for the advancement of the tooth, which at once takes place. Under continued pressure this action is renewed again and again until the tooth has reached its desired position. While this is taking place on the advancing side quite an opposite condition prevails on the side from which advancement has taken place. There the fibrous tissue of the pericementum has been subjected to extreme tension; greater room has been provided for the accommodation of the nutrient vessels, and osteoblasts have been developed for the formation of bony material to add to the alveolar wall, and thus close the space caused by the movement of the tooth. While these processes of absorption and reproduction on opposite sides of the tooth have been going on coincidentally, their results have been very unequal, for the absorption of bone is a far more rapid process than its formation.

During the entire time of moving, and for a long time afterwards, the tension of the pericementum on the free side of the tooth is kept up to such an extent that, were the force of pressure or of retention renewed, the tooth would at once be drawn partly back into the space created by its movement.

The tendency is only finally overcome after the deposit of ossific matter in the alveolar socket has been sufficient to allow the pericementum to resume its normal thickness on that side of the tooth, when,. by virtue of the removal of the tension and support of the new bony tissue, the backward movement of the tooth is no longer possible.

While this process of reparative construction has been going on, the structures about the opposite side of the tooth have been adjusting themselves to the new condition. The pressure upon the tooth having ceased, no more bone is absorbed; any injury inflicted upon the pericementum by its continuous compression is repaired; the nerves and blood-vessels resume their normal functions; and the tooth in its new position becomes a far more useful member of the dental organism than it had been.

Having thus reviewed, perhaps not with sufficient brevity, the character of the structures involved and the physiological changes that take place in the movement of teeth, I propose now to call your attention to three important considerations involved in tooth-movement that have not as yet been as fully emphasized by writers upon this subject as they deserve to be.

*First: the character of power to be applied for the movement of teeth.*

In changing the position of a tooth in the mouth we are governed by the principles of applied mechanics, subject, of course, to physiological conditions. Power must be applied, and to obtain it we avail ourselves of the use of such substances as will yield it in proper degree, and fashion and arrange them in a way that will produce the best results. Of the many materials suitable for power-production in the mouth, each has its value, and each its advantages in certain cases. In some instances the steady and continuous traction of elastic rubber is most desirable; in others, the elasticity of vulcanite or some of the metals; while in others still, the more powerful and interrupted force of the screw will be required. The age of the patient, his physical condition, and the peculiarities of the case will all have to be considered in determining when and where one or the other shall be used.

Viewed in a physiological light, we do not believe that there is any objection to the use of either continuous or interrupted pressure. Each we hold to be equally good in its place. It is not so much a question of the character of the force applied as it is the quality of it. If a certain movement is difficult of accomplishment, on account of the firmness of the teeth or of opposing influences, we use the screw in some of its forms; not because by its use we obtain a period of rest after a period of action, but because it yields the greatest amount of power.

So too, in a simple movement, we may often accomplish our object most advantageously by the use of a rubber band, on account of the simplicity of its application and operation, and not because the character of its action is continuous.

As to the amount of power to be applied in any given case, that will depend upon the judgment of the operator and a proper trial. If found to be too great, it can be lessened; if too little, it may be increased. The progress of the case and the comfort of the patient will determine the matter. The amount of power suitable for cases in general cannot be reduced to a scale or formulated into the law.

What is needed in all cases is the maximum of movement combined with the minimum of pain. The discomfort of the patient is a far better indication of the application of excessive power than anything else can be.

The most successful practitioner in this line of work is he who recognizes the relative value of the different power-producing appliances, whose judgment, unwarped by bias, indicates which it is better to apply in any given case, and whose ingenuity can so contrive and arrange them that they will yield the best results to both patient and operator.

The second is that of persistent force in tooth eruption.

Each tooth has a definite position assigned it in the arch, and this position it will occupy unless prevented by accidental circumstances. In seeking its position it is guided and directed by that inscrutable law of nature which governs and controls the development of every part of our physical frame, and it is impelled in its course by that power or force of nature which lies back of every physical movement.

These facts are familiar to us all; but do we often stop to consider the quality or power of this impelling force? When there are no hinderances, the tooth in the course of its eruption glides so gently and easily into its position that we are apt not to realize the power that is behind it; but when there are obstacles in the way that tend to prevent or hinder the full or normal eruption of the tooth, we see nature putting forth her full powers in endeavoring to remove or overcome them. If the impediment be of such a character that nature cannot overcome it, the tooth must either become imprisoned or remain but partially erupted. More frequently, however, nature will not allow herself to be entirely defeated, but by continuing to exercise her power the tooth will be compelled to assume a position as near the normal one as possible. In this way irregularity of position in the human teeth generally occurs.

Singularly enough, this power, lying back of the erupting tooth, is greater in certain teeth than in others, and its greatest manifestation is found in connection with the cuspids.

The wonderful force sometimes exerted by these teeth in endeavoring to gain their true position in the arch may be well illustrated by a case in the practice of the writer. (See Fig. 1.)

Fig. 1.

The patient was a young lady, about fifteen years of age, in whose upper jaw a cuspid had erupted outside of the arch, causing an unsightly projection of the lip. All of the other teeth were properly aligned, but the bicuspids and molars, on the affected side, were somewhat in advance of their true positions, and there was consequently very little space in the arch for the accommodation of this cuspid. The first molar, on the same side, was badly decayed, so it was decided to extract it as a preliminary to making room for the misplaced tooth. An appliance was then attached to the second molar and second bicuspid, intended to draw the latter tooth backward. The patient left with the fixture in position, and did not return until eighteen months later, when it was noticed that both bicuspids had moved backward, and the cuspid occupied its proper position in the arch. (See Fig. 2.)

It transpired that the appliance, having caused some pain, was removed by the patient two days after it had been placed in

Fig. 2.

position, and the case neglected. The correction of the irregularity had been entirely accomplished by the cuspid forcing its way into place and crowding bicuspids backward in the effort.

This case shows how nature sometimes succeeds as a corrector of irregularities.

In view, therefore, of what nature is able to do and will do towards bringing about a harmonious relation of the teeth, would it not be well for us to assign her a more important part in the regulation of teeth than we have been in the habit of doing? So important an ally should not be underrated or overlooked.

If our patient be deformed by too close a bite, let us insert a plate in the roof of the mouth with which only the anterior teeth of the lower jaw can come in contact, and in a little while nature will elongate the posterior teeth sufficiently to give us any desired opening of the bite. If a cuspid remains but partially erupted, or lies outside of the arch, let us make room for it, and it will lose no time in assuming its proper position. And if some of the teeth are crowded and locked out of position during the earlier period of second dentition, as is frequently the case with the inferior incisors, let us wait and see what change nature will bring about through enlargement of the arch and persistent power before we interfere with our mechanical agencies.

The third and last consideration I have to offer is that of securing immobility of teeth after regulating.

A retaining appliance, to be in the highest degree efficient, should be as light and simple in construction as possible; it should be non-removable; it should afford the least opportunity for the retention of food or secretions; it should be as inconspicuous as possible, and, last and most important, it should hold the teeth firmly while they are becoming fixed in their new positions.

With these requirements in view, the writer was led, many years ago, to devise certain appliances for retaining teeth almost immovably. So far as he is aware, they are the only ones that meet all of the requirements enumerated; and although, in their present state of development, they are not applicable to nearly all the cases that come to our hands, they are adapted to so many of the simpler forms of irregularity as to make them almost indispensable. They are an outgrowth of the original idea of the Magill band, and consist of gold or platinum bands, either single or in couples, to which are attached spurs, bars, or wires, to lie in contact with or bear upon other teeth in such a way as to secure absolute rigidity of the teeth to be retained. They are secured in position by cementing the bands to teeth selected for the purpose.

Fig. 3.

Fig. 4.

In the models before you may be seen some of the many ways in which they are formed and applied. In Fig. 3, the band, with small bar attached to rest against adjoining teeth, holds in position a cuspid that had been moved inward into line; in Fig. 4 a band, with single spur, retains an incisor that had been rotated; still another, consisting of two bands joined at their points of contact, was used to retain two centrals that had been rotated in opposite directions. A modification (Fig. 5), consisting of two bands joined to an extension bar, held in position two incisors that had been

Fig. 5.

drawn inward to close a space caused by the loss of a tooth; while on another model (Fig. 6) you may notice two bands joined by a thin platinum wire to serve the same purpose as the preceding one.

After teeth have been moved into proper position and retained there by the herein-described appliances until new walls have been

Fig. 6.

formed about them and the osseous tissue becomes thoroughly calcified, the teeth will henceforth remain in their new positions without extraneous aid. The importance of properly retaining teeth after regulation can scarcely be overestimated, for in many cases, after all the care and labor of regulating, the good results have been lost by a too limited period of retention. The length of time required for holding teeth in position before permanent retention is assured varies with each case, but a shorter period than six months should never be allowed. It is, however, not only the length of time that has to be considered, but the character of the retention as well.

A broken bone, after being set, is retained in rigid splints by the surgeon, not only to assure proper apposition of the fractured ends, but also to enable the reparative process to go on more quickly. So, when new alveolar process is to be built up about teeth, it is very evident that this operation will proceed rapidly or otherwise in proportion as the teeth are firmly held.

Most retaining appliances are of such construction that they must necessarily be removed at times to free them from the *débris* of food and the accumulation of vitiated secretions. Every time such appliance is removed and replaced the teeth are necessarily more or less disturbed in their positions, and, if the appliance be removable by the patient, there is no way of preventing him from removing it at improper times, should he feel so disposed. These conditions constitute a valid objection to removable retainers.

The results secured by the above method of rigid retention have been so satisfactory as to fully confirm us in our preconceived idea that the principle was the correct one for use in all cases.

## Figures and Tables

**Fig. 1. f1:**
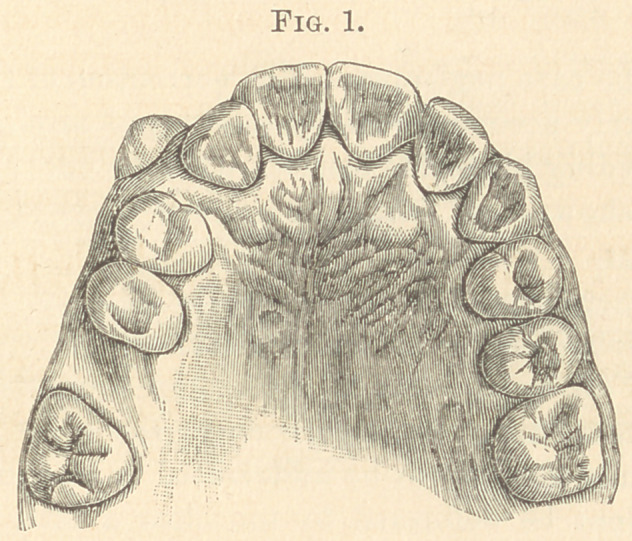


**Fig. 2. f2:**
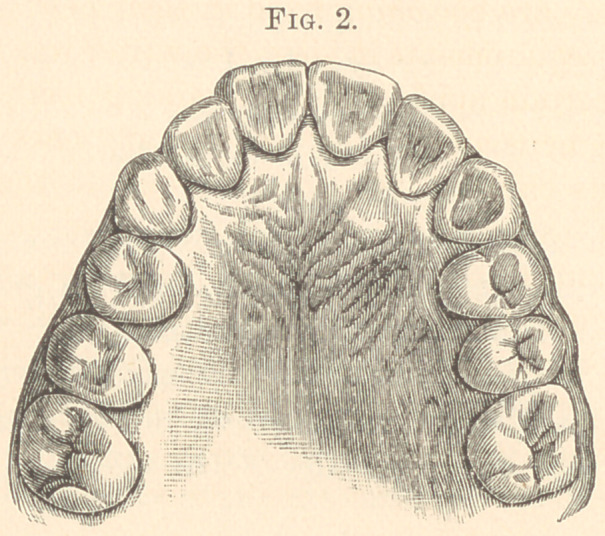


**Fig. 3. f3:**
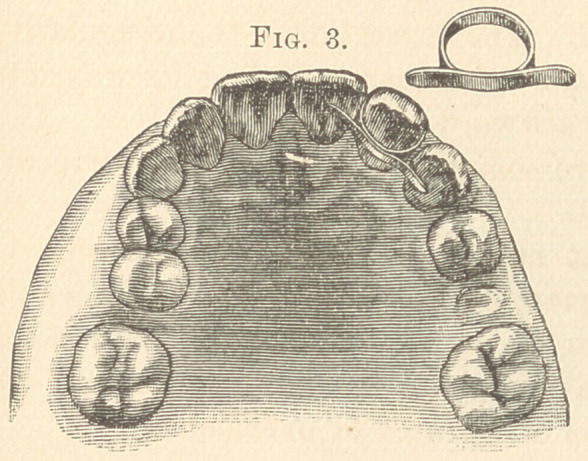


**Fig. 4. f4:**
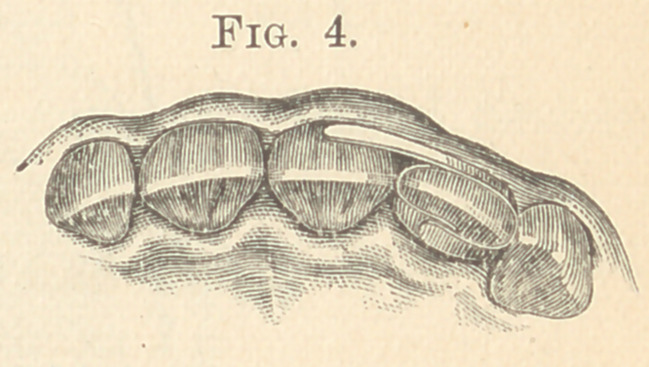


**Fig. 5. f5:**
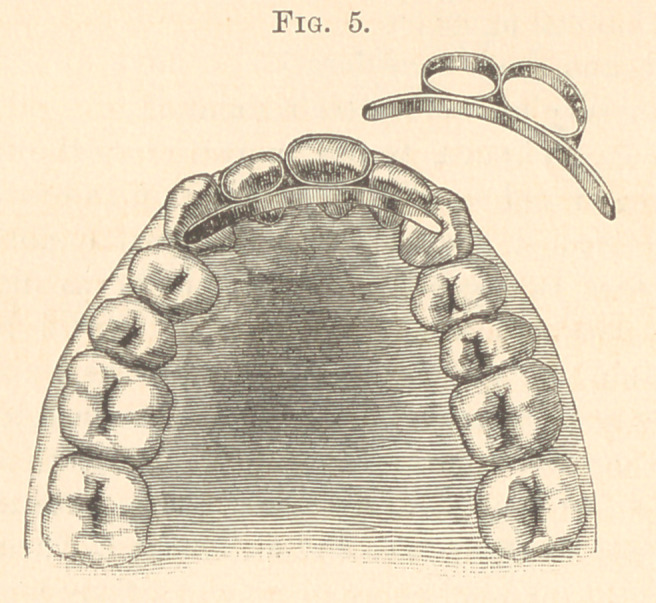


**Fig. 6. f6:**